# Treatment outcomes of pulp revascularization in traumatized immature teeth using calcium hydroxide and 2% chlorhexidine gel as intracanal medication

**DOI:** 10.1590/1678-7757-2020-0217

**Published:** 2020-09-25

**Authors:** Andrea Cardoso PEREIRA, Matheus Lima de OLIVEIRA, Ana Carolina C. L. CERQUEIRA-NETO, Brenda P. F. A. GOMES, Caio Cezar Randi FERRAZ, José Flávio Affonso de ALMEIDA, Marina Angélica MARCIANO, Adriana DE-JESUS-SOARES

**Affiliations:** 1 Universidade Estadual Campinas Faculdade de Odontologia de Piracicaba Departamento de Odontologia Restauradora PiracicabaSP Brasil Universidade Estadual Campinas - UNICAMP. Faculdade de Odontologia de Piracicaba. Departamento de Odontologia Restauradora, área de Endodontia. Piracicaba, SP, Brasil.; 2 Universidade Estadual Campinas Faculdade de Odontologia de Piracicaba Departamento de Diagnóstico Oral PiracicabaSP Brasil Universidade Estadual Campinas - UNICAMP. Faculdade de Odontologia de Piracicaba. Departamento de Diagnóstico Oral, área de Radiologia Oral, Piracicaba, SP, Brasil.

**Keywords:** Calcium hydroxide, Chlorhexidine, Endodontics, Regenerative endodontics, Tooth injuries

## Abstract

**Objective:**

Pulp revascularization is an effective treatment for immature necrotic teeth. Calcium hydroxide has been used in pulp revascularization as an intracanal medication due to its antimicrobial action and the non-exhibition of crown discoloration and cytotoxicity for stem cells from the apical papilla. Our study aimed to investigate the clinical success and quantitative radiographic changes of root development in immature traumatized teeth using calcium hydroxide plus 2% chlorhexidine gel as intracanal medication.

**Methodology:**

In this retrospective study, 16 patients were treated with a standardized pulp revascularization protocol. Calcium hydroxide and 2% chlorhexidine gel were manipulated in a 1:1 (v/v) ratio and inserted into root canals with Lentulo spirals (Dentsply Maillefer, Baillaigues, Switzerland). Patients were followed up for a period from 9 to 36 months for the evaluation of clinical and radiological data. Radiographic measurements of root length, root width, apical diameter, and MTA placement from the apex were quantified using software ImageJ. Wilcoxon test and t-test were used, according to nonparametric or parametric data, respectively, for changes over time in root length, root width, and apical diameter.

**Results:**

Fifteen teeth survived during the follow-up period (93.75%) and met the criteria for clinical success. Although the changes seem to be very small in many cases, significant increases in the average root length (14.28%, p<0.0001), root width (8.12%, p=0.0196), and decrease in apical diameter (48.37%, p=0.0007) were observed. MTA placement from the apex and age at the time of treatment was not significantly associated with the quantitative radiographic outcomes.

**Conclusions:**

Pulp revascularization in traumatized immature teeth treated with calcium hydroxide plus 2% chlorhexidine gel as intracanal medication had high success and survival rates, showing periodontal healing and resolution of signs and symptoms. However, concerning the continued root development, the outcomes can still be considered unpredictable.

## Introduction

Traumatic dental injuries mostly occur in kids between 7 and 14 years old, and pulp necrosis with interruption of root development is one of the main sequelae.^1,2^ If an injury occurs to an immature tooth without causing necrosis, reinnervation and reestablishment of the vascular supply are expected, allowing the tooth to continue its growth.^2^ However, if the pulp tissue becomes necrotic, blood supply is interrupted and, consequently, root development is suspended, resulting in a tooth with open apices and thin and fragile dentinal walls.^2,3^ The treatments proposed for this condition include a) apexification with periodic changes of calcium hydroxide-based medications; b) placement of an apical barrier with MTA (mineral trioxide aggregate), followed by root canal obturation with gutta-percha, and c) pulp revascularization.^3^

Pulp revascularization has been consolidated as a viable and effective alternative for the treatment of immature necrotic teeth.^3^ It has advantages over the conventional techniques, such as the possibility of continued root development and the consequent strengthening of the dental structure.^3,4^ Its steps involve decontamination with root canal irrigants, insertion of intracanal medication, induction of a blood clot, and coronal sealing.^5^

For a few years, a triple antibiotic paste, described by Hoshino, et al.^6^ (1996) and composed of metronidazole, ciprofloxacin, and minocycline, was considered a “gold-standard” intracanal medication for pulp revascularization procedures, because antibiotics are usually effective against endodontic pathogens.^7^ However, antibiotics offer risks such as allergic reactions, bacterial resistance, difficulty of removal, and the possibility of crown discoloration.^7,8^ The recent guidelines of the American Association of Endodontists (AAE) and the European Society of Endodontology (ESE)^5,9^ recommend then the use of calcium hydroxide-based medications. A recent survey found that calcium hydroxide intracanal medications are preferred by 52.2% of endodontists for pulp revascularization in the United States.^10^

Two percent of chlorhexidine gel is an active vehicle that confers additional antimicrobial properties to calcium hydroxide.^11^ This combination acts as a physical and chemical barrier, offers pH around 13, has chlorhexidine’s substantivity and a higher antimicrobial action than calcium hydroxide alone, and is effective against a large spectrum of microorganisms, including Gram-positive and Gram-negative bacteria, yeasts, and fungi.^12-14^ The effectiveness of this association has been shown in pulp revascularization with satisfactory clinical and radiographic outcomes.^4,14-16^

Although dental trauma is the main etiology for pulp necrosis in immature teeth,^17^ only three studies have reported the clinical and radiographic outcomes of pulp revascularization in immature traumatized teeth, exclusively.^4,18,19^ Therefore, this retrospective study aimed to investigate the clinical success and quantitative radiographic changes of root development in immature traumatized teeth using the combination of calcium hydroxide and 2% chlorhexidine gel as intracanal medication.

## Methodology

### Selection of dental records

The study protocol was approved by the local research ethics committee (protocol number 57189016). This retrospective study was based on the assessment of the patient’s records. The dental records of patients who sought treatment for traumatic dental injuries, from 2012 to 2017, in a local dental trauma service, with immature necrotic teeth, were selected for this study. The cases selected had at least six-month follow-up, appropriate data, and well-processed radiographs that allow qualitative and quantitative assessments.

### Treatment protocol

Two endodontists, with more than two years of experience, performed the pulp revascularization procedures, using the standard protocol of the local dental trauma service, according to Nagata, et al.^4^ (2014). An informed consent form was obtained after initial examination. The teeth were anesthetized with local anesthesia (2% lidocaine with vasoconstrictor – Alphacaine; DFL, Rio de Janeiro, RJ, Brazil), isolated with a rubber dam, accessed, and slowly and carefully irrigated with 6% sodium hypochlorite, which was inactivated by 5% sodium thiosulfate, followed by saline solution and 2% chlorhexidine, which was neutralized by 5% Tween 80 and 0.07% soy lecithin.^20^ There was no mechanical instrumentation. The canal was dried with absorbent paper points. Then, an intracanal medication, consisting of the combination of calcium hydroxide (Biodinâmica, Ibiporã, PR, Brazil) and 2% chlorhexidine gel (Endogel, Itapetininga, SP, Brazil) in a 1:1, v/v ratio, was inserted into the root canals with Lentulo spirals (Dentsply Maillefer, Baillaigues, Switzerland) and placed 3 mm from the working length. Subsequently, the access cavity was double-sealed with a 2-mm layer of a temporary sealing material (Coltene-Whaledent, Langenau, Germany), followed by a resin-bonded restoration (Z250 Filtek; 3M ESPE, Sumaré, SP, Brazil). In the second visit, after 21 days, the teeth were anesthetized with local anesthesia (2% lidocaine with vasoconstrictor), isolated with a rubber dam, accessed, and the intracanal medication was removed using abundant irrigation with saline solution (10 mL), followed by 5 mL of 17% EDTA for 3 minutes, and then by 10 mL of saline solution. A manual K-file (Dentsply Maillefer, Baillaigues, Switzerland) was inserted 1-2mm beyond the root apex to stimulate bleeding, and a 2-mm layer of collagen fiber (CollaCote; Zimmer Dental, Carlsbad, CA, USA) was placed over the blood clot, followed by the insertion of a 3-mm white MTA (Angelus, Londrina, PR, Brazil). Finally, the access cavity was double-sealed with Coltosol (Coltene-Whaledent, Langenau, Germany) and resin-bonded restoration (Z250 Filtek; 3M ESPE, Sumaré, SP, Brazil).

### Success criteria

Clinical success was defined as the absence of any signs or symptoms (spontaneous pain, swelling, sinus tract, pain associated with palpation or percussion), normal tooth mobility, absence of periapical radiolucency and root resorption.^21^ Survival was defined as the tooth remaining in the arch during the follow-up period, and tooth extraction was considered a failure.^18^ The incidence of adverse events was also noted.^22^

### Radiographic analysis

The preoperative and follow-up radiographs were taken using the standardized paralleling technique, bite registration with condensation silicone impression and receptor-holding instruments. Conventional radiographs were chemically processed and scanned with HP Scanjet G4050 (Hewlett-Packard Development Co., Palo Alto, CA, USA), and digital radiographs were obtained with Apixia Digital Imaging (Apixia Dental, San Jose, CA, USA). All radiographic images were saved in TIFF format and transferred to software ImageJ (ImageJ 1.49v; US National Institutes of Health, Bethesda, MD, USA). Size #2 of an intraoral radiographic image in ImageJ was calibrated by adjusting the image to a total area of 30×40 mm. The calibration process allowed the measurement of changes in root development in a millimetric scale.

**Figure 1:**
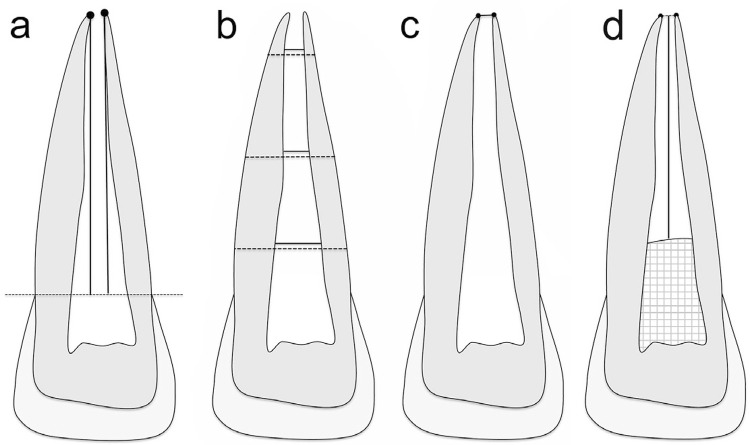
Measurements of radiographic images. (a) Root length: perpendicular line from the end of the root on the mesial and distal side to an imaginary line connecting both cementoenamel junctions; (b) Root width (dotted line) and pulp space (full line) at the apical, middle, and cervical third; (c) Apical diameter; (d) MTA placement from the apex

Under subdued room lighting, three examiners (two endodontists and one oral radiologist) marked, by consensus, the most apical point of the mesial and distal sides of the roots. Root length was measured using the straight-line tool from each apical point toward an imaginary line in the cementoenamel junction (Figure 1a). Root dentin width was measured at three levels of the root by subtracting the pulp space (full line; Figure 1b) from the root width (dotted line; Figure 1b). The root length was divided by four to standardize the location of the measurement levels in the apical, middle and cervical thirds. The measurements obtained at each level were averaged to define the overall width change. The diameter of the apical foramen was measured as the shortest distance between the most apical point of the mesial and distal sides of the roots (Figure 1c). MTA placement from the apex was measured as the shortest distance from the middle of the apex to the MTA barrier (represented as the vertical line; the squares represent the coronal sealing; Figure 1d). This measure was estimated in millimeters and associated with radiographic changes in root length, root width, and apical diameter.

The difference between preoperative and final follow-up radiographs was estimated in millimeters. The percentage of increase in root length, root width and decrease in apical diameter was calculated according to Nagy, et al.^23^(2014).

The measurements were conducted by the same examiner and repeated after 1 week. The mean of the two replicates was considered as the final value. Using the visual assessment method, the examiners evaluated the presence or absence of periapical radiolucency; signs of root resorption; intracanal calcification; and Cvek’s stage of root development,^24^ which varies between 1 and 5, whereas stages 1, 2 and 3 includes teeth with wide, divergent apical opening and a root length estimated to less than 1/2, 1/2, and 2/3 of the final root length, respectively; stage 4 includes teeth with wide open apical foramen and nearly completed root length; stage 5 includes teeth with closed apical foramen and completed root development.

### Statistical analysis

The descriptive analyses were expressed as frequencies and percentages or the median, maximum, and minimum value for the patients’ demographics, baseline characteristics, and success criteria. The intraclass correlation coefficient (ICC) was conducted to assess intraexaminer reliability in the radiographic analysis. The preoperative and follow-up measurements of root length, root width, and decrease of apical diameter were expressed as mean±standard deviation when data were normally distributed and parametric t-test was used; the median, maximum value, and minimum value were expressed when nonparametric Wilcoxon test was used. Pearson’s (for normally distributed data) and Spearman’s (if normally assumption is not met) correlations were used for an association between MTA placement from the apex; age at the time of treatment; and initial stage of root development with quantitative radiographic measurements of root length, root width, and decrease in apical diameter. Statistical significance was set at p<0.05. All statistical analyses were conducted using BioEstat 5.3 (Instituto Mamirauá, Belém, PA, Brazil).

## Results

### Baseline study population's characteristics

Table 1 shows the characteristics of the study population. Fifteen patients with 16 traumatized immature teeth met the inclusion criteria. In this population, the age at the time of treatment ranged between 7 and 18 years old, with an average of 9 years old. Fall (43.75%) and crown fracture associated with extrusive luxation (43.75%) was the main etiology and type of trauma, respectively. The follow-up period ranged between 9 and 36 months, with a median of 18 months.

**Table 1:** Patients’ demographics and baseline characteristics

Variable	Pulp Revascularization (15 patients/16 teeth)
**Sex**	
Male	8 (53.33%)
Female	7 (46.67%)
Age at treatment*	8.5; 18; 7
**Tooth**	
Maxillary central incisor	15 (93.75%)
Maxillary lateral incisor	1 (6.25%)
**Etiology of trauma**	
Fall	7 (43.75%)
Bicycle	5 (31.25%)
Sports	3 (18.75%)
Physical aggression	1 (6.25%)
**Type of trauma**	
Crown fracture + subluxation	1 (6.25%)
Crown fracture + extrusive luxation	7 (43.75%)
Crown fracture + intrusive luxation	1 (6.25%)
Extrusive luxation	2 (12.5%)
Lateral luxation	2 (12.5%)
Reimplantation	3 (18.75%)
**Presence of signs and symptomsǂ**	10 (62.5%)
**Presence of periapical radiolucency**	2 (12.5%)
**Presence of root resorption**	6 (37.5%)
Initial Cvek’s stage of root development*	3; 4; 1
Follow-up periods (months)*	18; 36; 9

^ǂ^Including spontaneous pain, swelling, sinus tract, pain associated with palpation or percussion, and tooth mobility.

*Median; maximum value; minimum value.

### Clinical outcomes

The survival rate was 93.75% (15/16); the failure case was a patient that retraumatized the tooth treated and avulsed it. Our study had a 93.75% (15/16) clinical success rate (Table 2). No tooth required additional endodontic treatment during the follow-up period. The main adverse event was crown discoloration (5/16) followed by retraumatization of the same tooth (1/16). All cases of crown discoloration were subjected to internal bleaching. All cases showing signs of root resorption in the initial exam were stabilized in the follow-up. During the follow-up periods, none of the teeth regained pulpal sensitivity or exhibited any signs or symptoms. Blood clot was stimulated in all cases.

**Table 2:** Clinical outcomes and adverse events

Variable	Pulp Revascularization (n=16)
**Clinical success - n (%)**	15 (93.75%)
**Survival - n (%)**	15 (93.75%)
**Adverse events n (%)**	6 (37.5%)
Crown discoloration	5 (83.33%)
Retraumatized	1 (16.67%)

### Radiographic outcomes

Table 3 shows the radiographic outcomes. There was an excellent intraexaminer agreement for the radiographic measurements, with a 0.935 mean ICC value. Only the cases that survived the follow-up period were included in these analyses (15/16).

**Table 3:** Quantitative radiographic outcomes

Radiographic measurement	Stage	Mean±SD (mm)/Median, maximum value, minimum value (mm)	P-value
**Increase in root length**	**Preoperative**	12.25±2.19	<0.0001
	**Follow-up**	13.85±2.17	
**Increase in root width**	**Preoperative**	3.21±0.29	0.0196
	**Follow-up**	3.46±0.36	
Apical third	**Preoperative**	2.54±0.29	0.0011
	**Follow-up**	2.93±0.5	
Middle third	**Preoperative**	3.42; 3.9; 2.87	0.1641
	**Follow-up**	3.5; 4.98; 3.01	
Cervical third	**Preoperative**	3.75±0.58	0.9729
	**Follow-up**	3.75±0.6	
**Decrease in apical diameter**	**Preoperative**	2.1; 3.75; 1.03	0.0007
	**Follow-up**	0.97; 2.19; 0.63	

T-test was used for values expressed as mean±SD; Wilcoxon test was used for values expressed as median, maximum value, and minimum value.

**Figure 2:**
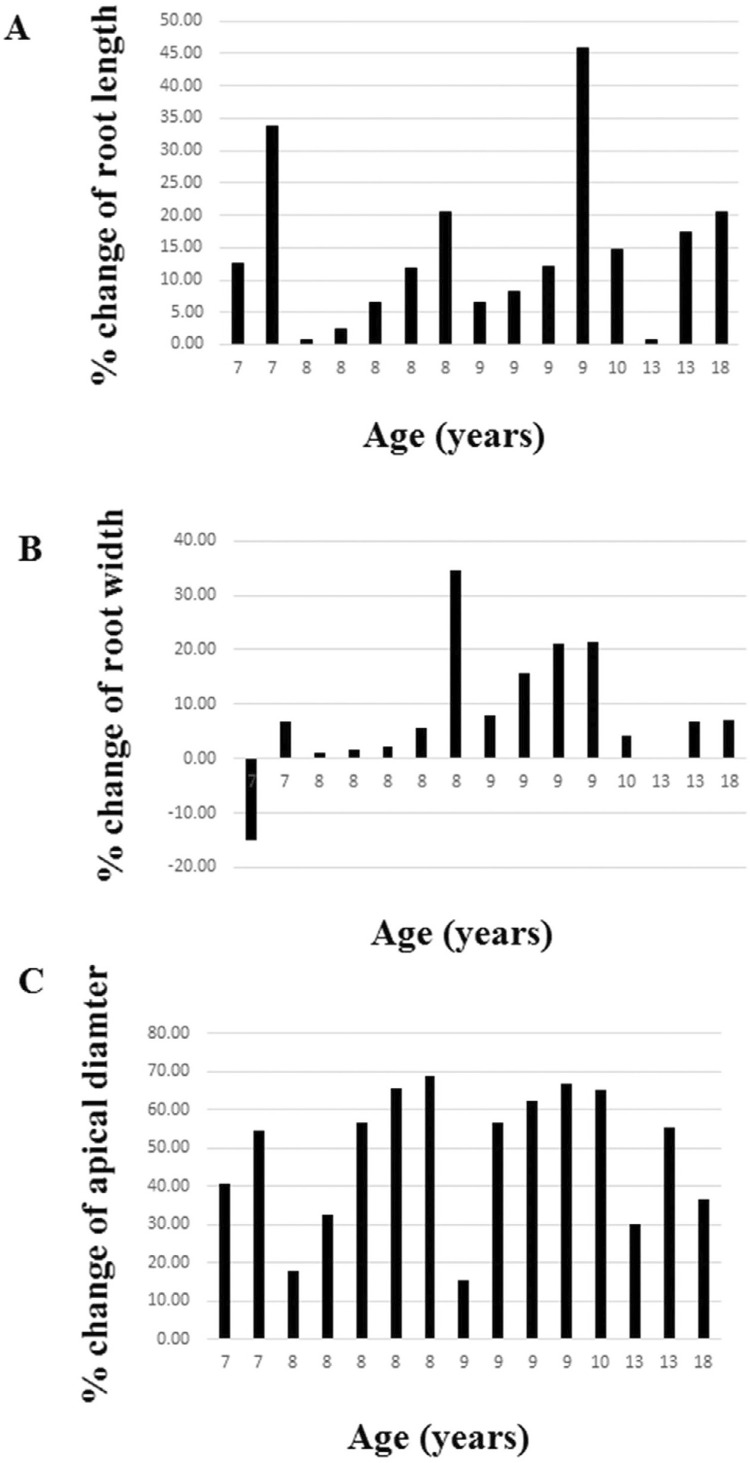
Patient’s age and radiographic changes in (A) increase in root length; (B) increase in root width; (C) decrease in apical diameter

A significant increase in root length, root width, and decrease in apical diameter were observed from the preoperative radiographs to the follow-up period. The apical third was the only one that increased significantly; however, it presented no statistical difference when compared with the percentage increase among the three thirds (p>0.05; Kruskal-Wallis test). The average increase in root length, root width, and decrease in apical diameter was 14.28%, 8.12%, and 48.37%, respectively. Assuming a 20% difference as clinical radiographic change,^18,21,22,25^ 4 teeth (26.67%) achieved the criteria for root length, 3 teeth (20%) for root width, and 13 teeth (86.67%) for decrease in apical diameter, which ranged from 15.54% to 68.8% (Figure 2). MTA placement from the apex and age at the time of treatment was not significantly associated with the radiographic outcomes of continued root development. The stage of root development was not associated with a higher percentage of change in root length and root width, only with a decrease in apical diameter. Two cases presented intracanal calcification.

**Figure 3:**
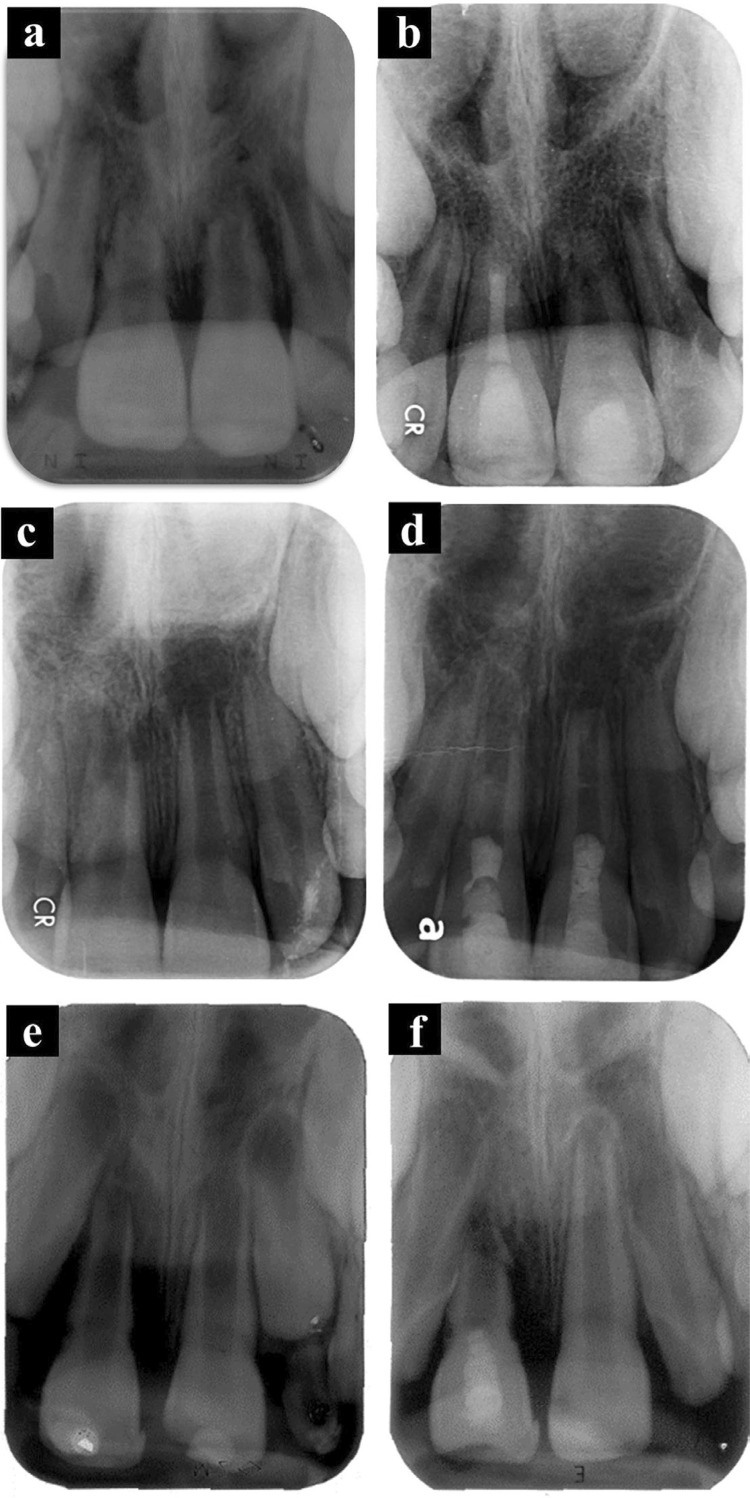
(a) Initial radiography of a 9-year old girl with extrusive luxation of the maxillary left central incisor after a fall; (b) After 24 months, showing continued root development. (c) Initial radiography of a 9-year old boy with extrusive luxation of the right and left maxillary central incisors after sports activity. This radiography showed signs of root resorption. This patient took 3 years to seek treatment in the dental trauma service, after receiving initial care in a hospital; (d) After 18 months, showing periapical healing, incomplete apical closure, absence of a significant increase in root length and root width, and stabilization of root resorption. (e) Preoperative radiograph. (f) Follow-up radiograph 9 months after treatment showing replacement resorption. Subsequently, the patient retraumatized the tooth, and it was avulsed

Figure 3 shows the periapical radiographs of representative clinical cases included in this study.

## Discussion

Our study shows the clinical and radiographic outcomes of pulp revascularization in traumatized immature teeth, after the use of calcium hydroxide with 2% chlorhexidine gel as intracanal medication. Despite its retrospective design, only two operators performed all cases by the standard protocol, being supervised by the coordinator of the dental trauma service in which the study was conducted. We included only the clinical dental records with complete data and that allowed the evaluation of all parameters reported in the study. The follow-up period ranged between 9 and 36 months. The lack of standardization of follow-up periods was a limitation of our study that could have influenced the results. However, obtaining a longer follow-up period was difficult in certain patients. The follow-up period of 9 months just happened in one case due to a reported failure. Most of teeth with clinical success had a follow up of 18 months, which can be considered an adequate time to observe radiographic changes in root development and resolution of periapical radiolucency.^9^The use of standardized radiographs with bite registration, receptor-holding instruments, and the same ray machine were fundamental to allow the quantification of changes in root length, root width, and apical diameter. Moreover, by the analysis of the clinical records, we could verify aspects such as type and etiology of dental trauma, presence of root resorption and periapical radiolucency, signs, and symptoms, adverse events, and clinical success rates.

The clinical success rate in our study was 93.75%. This result is similar to those of studies that reported from 76% to 100% clinical success rates.^4,18,21,25-27^ This finding confirms that pulp revascularization is a viable and effective procedure for immature traumatized teeth, with satisfactory results regarding the resolution of clinical and radiographic signs and symptoms. The most frequent adverse event was crown discoloration (5/16 cases), which is also observed in several studies.^4,19,21,22,26^ Despite the use of a calcium hydroxide-based intracanal medication, discoloration could not be avoided, probably due to the use of white MTA,^8^ even if this material is carefully inserted below the cementoenamel junction. This crown discoloration can be attributed to the presence of bismuth oxide in white MTA, which interacts with collagen in the dentin matrix, and migration of these ions to dentinal tubules, culminating in color change even in distant areas.^28^ All cases of crown discoloration observed during the follow-up were subjected to internal dental bleaching, with favorable aesthetic results. Achieving an adequate coronal seal is essential, since pulp revascularization is efficient only in teeth with the absence of root canal infection, that is, placement of an adequate material is crucial to avoid bacterial invasion into the pulp canal space.^29^ MTA is the most material used for coronal barrier^10^ due to its beneficial properties, as good sealing ability, low cytotoxicity, antimicrobial effect, biocompatibility and ability to induce proliferation of stem cells from apical papilla.^30^

The most frequently observed radiographic finding was decreased in apical diameter, ranging from 15.54% to 68.8%, which is following with several studies that consider it the most consistent and significant radiographic finding.^4,18,19,21,25,27^ More immature developmental teeth, such as Cvek’s stages one, two, and three,^24^ achieved the highest rates of decrease in apical diameter, confirming that pulp revascularization is an efficient procedure in traumatized teeth at the early stages of root development.

The second most frequently observed radiographic finding was increased root length, ranging from 0.74% to 45.88%, with an average of 14.28%. Considering the threshold of a 20% increase in radiographic outcomes as cutoff point,^18,21,22,25^ only 4 cases met this criterion. In the studies by Saoud, et al.^18^(2014), Chan, et al.^21^(2017), and Alobaid, et al.^22^ (2014), no cases met this criterion, whereas in the study by Li, et al.^25^(2017), in which dens evaginatus was the only etiology, 11 cases achieved this result. Despite the possible damage that luxation injuries exert on Hertwig’s epithelial root sheath^17^, the rates of increase in root length are similar to those of studies that involved several etiologies (ranging from 8.6% to 23.37%).^21-23,25-27^ However, it is slightly higher compared to studies that only included dental traumas.^18,19^

The third most frequently observed radiographic finding was increased root width. The apical third presented a greater increase in width, possibly because of the greater invagination of stem cells of the apical papilla in this region. However, only three cases had meaningful radiographic changes, whereas Saoud, et al.^18^ (2014) found nine cases (45%) that met this criterion. The average increase in root width was 8.12%, being comparable to Nazzal, et al.^19^ (2018) study, but slightly lower than the average of similar studies, in which this finding ranged from 13.75% to 28.2%.^18,22,27,31,32^ This finding suggests that root strengthening in terms of width is unpredictable. Despite the significant statistical results regarding the increase in root length and width, these changes cannot be considered clinically consistent, since in most cases they are quite small and only observed by radiographic measurements.

In our study, most traumatic injuries were extrusive luxations, associated or not with enamel; enamel and dentin; or enamel, dentin, and pulp fractures. Extrusive luxation is considered a moderate trauma to the supporting tissue and, when associated with crown fractures, the chance of pulp necrosis is around 40% in immature teeth, a lower rate compared to the estimate of pulp necrosis in lateral luxation (50%) or intrusions (100%).^2^These findings differ from previous studies conducted only in traumatized immature teeth, in which most injuries were lateral luxations;^4^ enamel, dentin, and pulp fractures;^18^or enamel and dentin fractures.^19^Severe traumas such as intrusions and avulsions were also found in smaller proportions, as well as in studies in which dental trauma was not the only etiology.^21,22^ Avulsion followed by replantation was observed in three cases: two with clinical success and one that failed because of new trauma. One of the factors that contributed to clinical success was the favorable conditions of replantation: both had a saline solution as a storage medium and were replanted within 60 minutes, according to IADT guidelines.^33^ The tooth in the failure case was replanted 19 hours after the avulsion and already exhibited signs of replacement resorption before retraumatization. This shows that pulp revascularization may be considered a viable treatment option when replantation is performed under favorable conditions.

In all cases of our study, the professionals used local anesthesia with vasoconstrictor, according to the protocol of Nagata, et al.^4^ (2014). Bleeding was more difficult to be achieved in certain patients and easier in others. However, the intracanal bleeding was achieved in all cases. Although most clinical studies in pulp revascularization recommend the use of local anesthesia without vasoconstrictor, the ESE guidelines^5^ reported that the evidence of improved bleeding without vasoconstrictor is scarce.

The disinfection of the root canals was performed with sodium hypochlorite and chlorhexidine, as passive decontamination, without mechanical instrumentation to preserve the root canal walls, according to the clinical protocol of Nagata, et al.^4^ (2014). The guidelines of AAE^9^ and ESE^5^do not recommend mechanical instrumentation. The concentration of sodium hypochlorite used may be considered high. Nevertheless, there was no consensus on the adequate concentration of this irrigant by the time this clinical research started. Currently, AAE^9^ and ESE^5^recommend the concentration varying between 1.5-3%. However, in the protocol used in our study, the irrigating solutions (sodium hypochlorite and chlorhexidine) were inserted approximately 3 mm below the working length to prevent injury to the stem cells of the apical papilla. Moreover, both irrigants were neutralized to decrease their cytotoxicity to the stem cells if in contact with them. Two percent chlorhexidine was neutralized with Tween 80 and soy lecithin and 6% sodium hypochlorite with sodium thiosulfate. Although chlorhexidine concentrations of 2% had a detrimental effect on the survival of stem cells, this effect can be reversed by a short irrigation time and subsequent application of neutralizing agents, such as Tween and lecithin.^34^To complement the disinfection of the root canals, calcium hydroxide associated with 2% chlorhexidine gel was the only intracanal medication used. This association allows the increase of antimicrobial activity against some bacteria found in endodontic infections and diffusion into dentinal tubules, without interfering in the chemical and biological properties of calcium hydroxide.^35,36^ Some studies have succeeded with the use of this medication in pulp revascularization,^4,14-16^ achieving similar results to triple antibiotic paste.^4,15^ For some time, the use of calcium hydroxide in pulp revascularization was not indicated due to its high pH and the possible tissue necrosis, preventing the differentiation of mesenchymal cells into new vital tissue.^30^ However, more recent studies showed that calcium hydroxide can be recommended in pulp revascularization.^5,37,38^ In addition to its property of microbial reduction and to the fact that it does not have the potential for crown discoloration, it also does not exhibit cytotoxicity to stem cells of the apical papilla, increases the release of dentin growth factors by EDTA, provides a better environment for attachment of viable apical papilla cells on dentin and does not cause bacterial resistance, as it can happen if there is a random use of antibiotics.^5,37-39^ Clinical studies and case series have also shown the clinical success of pulp revascularization with the use of calcium hydroxide-based intracanal medications.^27,32,40^

## Conclusions

Our results suggest that pulp revascularization with the combination of calcium hydroxide and 2% chlorhexidine gel as the intracanal medication is a viable treatment for traumatized immature teeth, presenting high success rates (93.75%), periodontal healing, and resolution of signs and symptoms. However, concerning continued root development, the outcomes can still be considered unpredictable, as only a few cases achieved a satisfactory root development.

## References

[1] - Diangelis AJ, Andreasen JO, Ebeleseder KA, Kenny DJ, Trope M, Sigurdsson A, et al. International Association of Dental Traumatology guidelines for the management of traumatic dental injuries: 1. Fractures and luxations of permanent teeth. Dent Traumatol. 2012;28(1):2-12. doi: 10.1111/j.1600-9657.2011.01103.x 10.1111/j.1600-9657.2011.01103.x 22230724

[2] - Andreasen FM, Kahler B. Pulpal response after acute dental injury in the permanent dentition: clinical implications-a review. J Endod. 2015;41(3):299-308. doi: 10.1016/j.joen.2014.11.015 10.1016/j.joen.2014.11.015 25601716

[3] - Chen YP, Jovani-Sancho MM, Sheth CC. Is revascularization of immature permanent teeth an effective and reproducible technique? Dent Traumatol. 2015;31(6):429-36. doi: 10.1111/edt.12214 10.1111/edt.12214 26370158

[4] - Nagata JY, Gomes BP, Rocha Lima TF, Murakami LS, Faria DE, Campos GR, et al. Traumatized immature teeth treated with 2 protocols of pulp revascularization. J Endod. 2014;40(5):606-12. doi: 10.1016/j.joen.2014.01.032 10.1016/j.joen.2014.01.032 24767551

[5] - Galler KM, Krastl G, Simon S, Van Gorp G, Meschi N, Vahedi B, et al. European Society of Endodontology position statement: revitalization procedures. Int Endod J. 2016;49(8):717-23. doi: 10.1111/iej.12629 10.1111/iej.12629 26990236

[6] 6 - Hoshino E, Kurihara-Ando N, Sato I, Uematsu H, Sato M, Kota K, et al. *In vitro* antibacterial susceptibility of bacteria taken from infected root dentine to a mixture of ciprofloxacin, metronidazole and minocycline. Int Endod J. 1996;29(2):125-30. doi: 10.1111/j.1365-2591.1996.tb01173.x 10.1111/j.1365-2591.1996.tb01173.x 9206436

[7] - Montero-Miralles P, Martín-González J, Alonso-Ezpeleta O, Jiménez Sanchéz MC, Velasco-Ortega E, Segura-Egea JJ. Effectiveness and clinical implications of the use of topical antibiotics in regenerative endodontic procedures: a review. Int Endod J. 2018;51(9):981-8. doi: 10.1111/iej.12913 10.1111/iej.12913 29480932

[8] - Kahler B, Rossi-Fedele G. A review of tooth discoloration after regenerative endodontic therapy. J Endod. 2016;42(4):563-9. doi: 10.1016/j.joen.2015.12.022 10.1016/j.joen.2015.12.022 26852148

[9] 9 - American Association of Endodontics. AAE Clinical Considerations for a Regenerative Procedure [internet] [cited 2020 July 24]. Chicago, Il; 2018. Available from: https://www.aae.org/specialty/clinical-resources/regenerative-endodontics/considerationsforregendo_asofapril2018-3/

[10] - Lee JY, Kersten DD, Mines P, Beltran TA. Regenerative endodontic procedures among endodontists: a web-based survey. J Endod. 2018;44(2):250-5. doi: 10.1016/j.joen.2017.09.010 10.1016/j.joen.2017.09.010 29229459

[11] - Signoretti FG, Gomes BP, Montagner F, Barichello Tosello F, Jacinto RC. Influence of 2% chlorhexidine gel on calcium hydroxide ionic dissociation and its ability of reducing endotoxin. Oral Surg Oral Med Oral Pathol Oral Radiol Endod. 2011;111(5):653-8. doi: 10.1016/j.tripleo.2010.11.016 10.1016/j.tripleo.2010.11.016 21393032

[12] - Gomes BP, Vianna ME, Zaia AA, Almeida JF, Souza-Filho FJ, Ferraz CC. Chlorhexidine in endodontics. Braz Dent J. 2013;24(2):89-102. doi: 10.1590/0103-6440201302188 10.1590/0103-6440201302188 23780357

[13] 13 - Gomes BP, Souza SF, Ferraz CC, Teixeira FB, Zaia AA, Valdrighi L, et al. Effectiveness of 2% chlorhexidine gel and calcium hydroxide against Enterococcus faecalis in bovine root dentine *in vitro*. Int Endod J. 2003;36(4):267-75. doi: 10.1046/j.1365-2591.2003.00634.x 10.1046/j.1365-2591.2003.00634.x 12702121

[14] - Soares AJ, Lins FF, Nagata JY, Gomes BP, Zaia AA, Ferraz CC, et al. Pulp revascularization after root canal decontamination with calcium hydroxide and 2% chlorhexidine gel. J Endod. 2013;39(3):417-20. doi: 10.1016/j.joen.2012.10.005 10.1016/j.joen.2012.10.005 23402520

[15] - Nagata JY, Soares AJ, Souza-Filho FJ, Zaia AA, Ferraz CC, Almeida FJ, et al. Microbial evaluation of traumatized teeth treated with triple antibiotic paste or calcium hydroxide with 2% chlorhexidine gel in pulp revascularization. J Endod. 2014;40(6):778-83. doi: 10.1016/j.joen.2014.01.038 10.1016/j.joen.2014.01.038 24862703

[16] - Nagata JY, Rocha-Lima TF, Gomes BP, Ferraz CC, Zaia AA, Souza-Filho FJ, et al. Pulp revascularization for immature replanted teeth: a case report. Aust Dent J. 2015;60(3):416-20. doi: 10.1111/adj.12342 10.1111/adj.12342 26219350

[17] - Diogenes A, Ruparel NB. Regenerative endodontic procedures: clinical outcomes. Dent Clin North Am. 2017;61(1):111-25. doi: 10.1016/j.cden.2016.08.004 10.1016/j.cden.2016.08.004 27912813

[18] - Saoud TM, Zaazou A, Nabil A, Moussa S, Lin LM, Gibbs JL. Clinical and radiographic outcomes of traumatized immature permanent necrotic teeth after revascularization/revitalization therapy. J Endod. 2014;40(12):1946-52. doi: 10.1016/j.joen.2014.08.023 10.1016/j.joen.2014.08.023 PMC4451000 25443280

[19] - Nazzal H, Kenny K, Altimimi A, Kang J, Duggal MS. A prospective clinical study of regenerative endodontic treatment of traumatized immature teeth with necrotic pulps using bi-antibiotic paste. Int Endod J. 2018;51(Suppl 3):e204-15. doi: 10.1111/iej.12808 10.1111/iej.12808 28653761

[20] - Siqueira JF Jr, Silva CH, Cerqueira MD, Lopes HP, Uzeda M. Effectiveness of four chemical solutions in eliminating Bacillus subtilis spores on gutta-percha cones. Endod Dent Traumatol. 1998;14(3):124-6. 10.1111/j.1600-9657.1998.tb00824.x 9863421

[21] - Chan EK, Desmeules M, Cielecki M, Dabbagh B, Ferraz dos Santos B. Longitudinal cohort study of regenerative endodontic treatment for immature necrotic permanent teeth. J Endod. 2017;43(3):395-400. doi: 10.1016/j.joen.2016.10.035 10.1016/j.joen.2016.10.035 28110920

[22] - Alobaid AS, Cortes LM, Lo J, Nguyen TT, Albert J, Abu-Melha AS, et al. Radiographic and clinical outcomes of the treatment of immature permanent teeth by revascularization or apexification: a pilot retrospective cohort study. J Endod. 2014;40(8):1063-70. doi: 10.1016/j.joen.2014.02.016 10.1016/j.joen.2014.02.016 PMC4159254 25069909

[23] - Nagy MM, Tawfik HE, Hashem AA, Abu-Seida AM. Regenerative potential of immature permanent teeth with necrotic pulps after different regenerative protocols. J Endod. 2014;40(2):192-8. doi: 10.1016/j.joen.2013.10.027 10.1016/j.joen.2013.10.027 24461403

[24] - Cvek M. Prognosis of luxated non-vital maxillary incisors treated with calcium hydroxide and filled with gutta-percha. A retrospective clinical study. Endod Dent Traumatol. 1992;8(2):45-55. 10.1111/j.1600-9657.1992.tb00228.x 1521505

[25] - Li L, Pan Y, Mei L, Li J. Clinical and radiographic outcomes in immature permanent necrotic evaginated teeth treated with regenerative endodontic procedures. J Endod. 2017;43(2):246-51. doi: 10.1016/j.joen.2016.10.015 10.1016/j.joen.2016.10.015 27955921

[26] - Kahler B, Mistry S, Moule A, Ringsmuth AK, Case P, Thomson A, et al. Revascularization outcomes: a prospective analysis of 16 consecutive cases. J Endod. 2014;40(3):333-8. doi: 10.1016/j.joen.2013.10.032 10.1016/j.joen.2013.10.032 24565648

[27] - Linsuwanont P, Sinpitaksakul P, Lertsakchai T. Evaluation of root maturation after revitalization in immature permanent teeth with nonvital pulps by cone beam computed tomography and conventional radiographs. Int Endod J. 2017;50(9):836-46. doi: 10.1111/iej.12705 10.1111/iej.12705 27689773

[28] - Marciano MA, Duarte MA, Camilleri J. Dental discoloration caused by bismuth oxide in MTA in the presence of sodium hypochlorite. Clin Oral Investig. 2015;19(9):2201-9. doi: 10.1007/s00784-015-1466-8 10.1007/s00784-015-1466-8 25922130

[29] - Aly MM, Taha SE, El Sayed MA, Youssef R, Omar HM. Clinical and radiographic evaluation of Biodentine and Mineral Trioxide Aggregate in revascularization of non-vital immature permanent anterior teeth (randomized clinical study). Int J Paediatr Dent. 2019;29(4):464-73. doi:10.1111/ipd.12474 10.1111/ipd.12474 30702789

[30] - Staffoli S, Plotino G, Nunez Torrijos BG, Grand NM, Bossù M, Gambarini G, et al. Regenerative endodontic procedures using contemporary endodontic materials. Materials (Basel). 2019;12(6):908. doi:10.3390/ma12060908 10.3390/ma12060908 PMC6471897 30893790

[31] - Jeeruphan T, Jantarat J, Yanpiset K, Suwannapan L, Khewsawai P, Hargreaves KM. Mahidol study 1: comparison of radiographic and survival outcomes of immature teeth treated with either regenerative endodontic or apexification methods: a retrospective study. J Endod. 2012;38(10):1330-6. doi: 10.1016/j.joen.2012.06.028 10.1016/j.joen.2012.06.028 22980172

[32] - Silujjai J, Linsuwanont P. Treatment outcomes of apexification or revascularization in nonvital immature permanent teeth: a retrospective study. J Endod. 2017;43(2):238-45. doi: 10.1016/j.joen.2016.10.030 10.1016/j.joen.2016.10.030 28132710

[33] - Andersson L, Andreasen JO, Day P, Heithersay G, Trope M, Diangelis AJ, et al. Association of Dental Traumatology guidelines for the management of traumatic dental injuries: 2. Avulsion of permanent teeth. Dent Traumatol. 2012;28(2):88-96. doi: 10.1111/j.1600-9657.2012.01125.x 10.1111/j.1600-9657.2012.01125.x 22409417

[34] - Widbiller M, Althumairy RI, Diogenes A. Direct and indirect effect of chlorhexidine on survival of stem cells from the apical papilla and its neutralization. J Endod. 2019;45(2):156-60. doi: 10.1016/j.joen.2018.11.012 10.1016/j.joen.2018.11.012 30711171

[35] 35 - Gomes BP, Vianna ME, Sena NT, Zaia AA, Ferraz CC, Souza-Filho FJ. *In vitro* evaluation of the antimicrobial activity of calcium hydroxide combined with chlorhexidine gel used as intracanal medicament. Oral Surg Oral Med Oral Pathol Oral Radiol Endod. 2006;102(4):544-50. doi: 10.1016/j.tripleo.2006.04.010 10.1016/j.tripleo.2006.04.010 16997123

[36] - Gomes BP, Montagner F, Berber VB, Zaia AA, Ferraz CC, Almeida JF, et al. Antimicrobial action of intracanal medicaments on the external root surface. J Dent. 2009;37(1):76-81. doi: 10.1016/j.jdent.2008.09.009 10.1016/j.jdent.2008.09.009 18995944

[37] - Galler KM, Buchalla W, Hiller KA, Federlin M, Eidt A, Schiefersteiner M, et al. Influence of root canal disinfectants on growth factor release from dentin. J Endod. 2015;41(3):363-8. doi: 10.1016/j.joen.2014.11.021 10.1016/j.joen.2014.11.021 25595468

[38] - Pereira TC, Vasconcelos LR, Graeff MS, Duarte MA, Bramante CM, Andrade FB. Intratubular disinfection with tri-antibiotic and calcium hydroxide pastes. Acta Odontol Scand. 2017;75(2):87-93. doi:10.1080/00016357.2016.1256427 10.1080/00016357.2016.1256427 27866468

[39] - Kitikuson P, Srisuwan T. Attachment ability of human apical papilla cells to root dentin surfaces treated with either 3mix or calcium hydroxide. J Endod. 2016;42(1):89-94. doi: 10.1016/j.joen.2015.08.021 10.1016/j.joen.2015.08.021 26454719

[40] - Cehreli ZC, Isbitiren B, Sara S, Erbas G. Regenerative endodontic treatment (revascularization) of immature necrotic molars medicated with calcium hydroxide: a case series. J Endod. 2011;37(9):1327-30. doi: 10.1016/j.joen.2011.05.033 10.1016/j.joen.2011.05.033 21846559

